# Aging-related biomarker discovery in the era of immune checkpoint inhibitors for cancer patients

**DOI:** 10.3389/fimmu.2024.1348189

**Published:** 2024-03-15

**Authors:** Abdullah Al-Danakh, Mohammed Safi, Yuli Jian, Linlin Yang, Xinqing Zhu, Qiwei Chen, Kangkang Yang, Shujing Wang, Jianjun Zhang, Deyong Yang

**Affiliations:** ^1^ Department of Urology, First Affiliated Hospital of Dalian Medical University, Dalian, Liaoning, China; ^2^ Department of Thoracic/Head and Neck Medical Oncology, The University of Texas MD Anderson Cancer Center, Houston, TX, United States; ^3^ Department of Biochemistry and Molecular Biology, Institute of Glycobiology, Dalian Medical University, Dalian, China; ^4^ Institute for Genome Engineered Animal Models of Human Diseases, National Center of Genetically Engineered Animal Models for International Research, Dalian Medical University, Dalian, Liaoning, China; ^5^ Department of Surgery, Healinghands Clinic, Dalian, Liaoning, China

**Keywords:** aging, immunosenescence, neoplasm, immune biomarkers, immune checkpoint inhibitors

## Abstract

Older patients with cancer, particularly those over 75 years of age, often experience poorer clinical outcomes compared to younger patients. This can be attributed to age-related comorbidities, weakened immune function, and reduced tolerance to treatment-related adverse effects. In the immune checkpoint inhibitors (ICI) era, age has emerged as an influential factor impacting the discovery of predictive biomarkers for ICI treatment. These age-linked changes in the immune system can influence the composition and functionality of tumor-infiltrating immune cells (TIICs) that play a crucial role in the cancer response. Older patients may have lower levels of TIICs infiltration due to age-related immune senescence particularly T cell function, which can limit the effectivity of cancer immunotherapies. Furthermore, age-related immune dysregulation increases the exhaustion of immune cells, characterized by the dysregulation of ICI-related biomarkers and a dampened response to ICI. Our review aims to provide a comprehensive understanding of the mechanisms that contribute to the impact of age on ICI-related biomarkers and ICI response. Understanding these mechanisms will facilitate the development of treatment approaches tailored to elderly individuals with cancer.

## 1 Introduction

**Figure 1:**
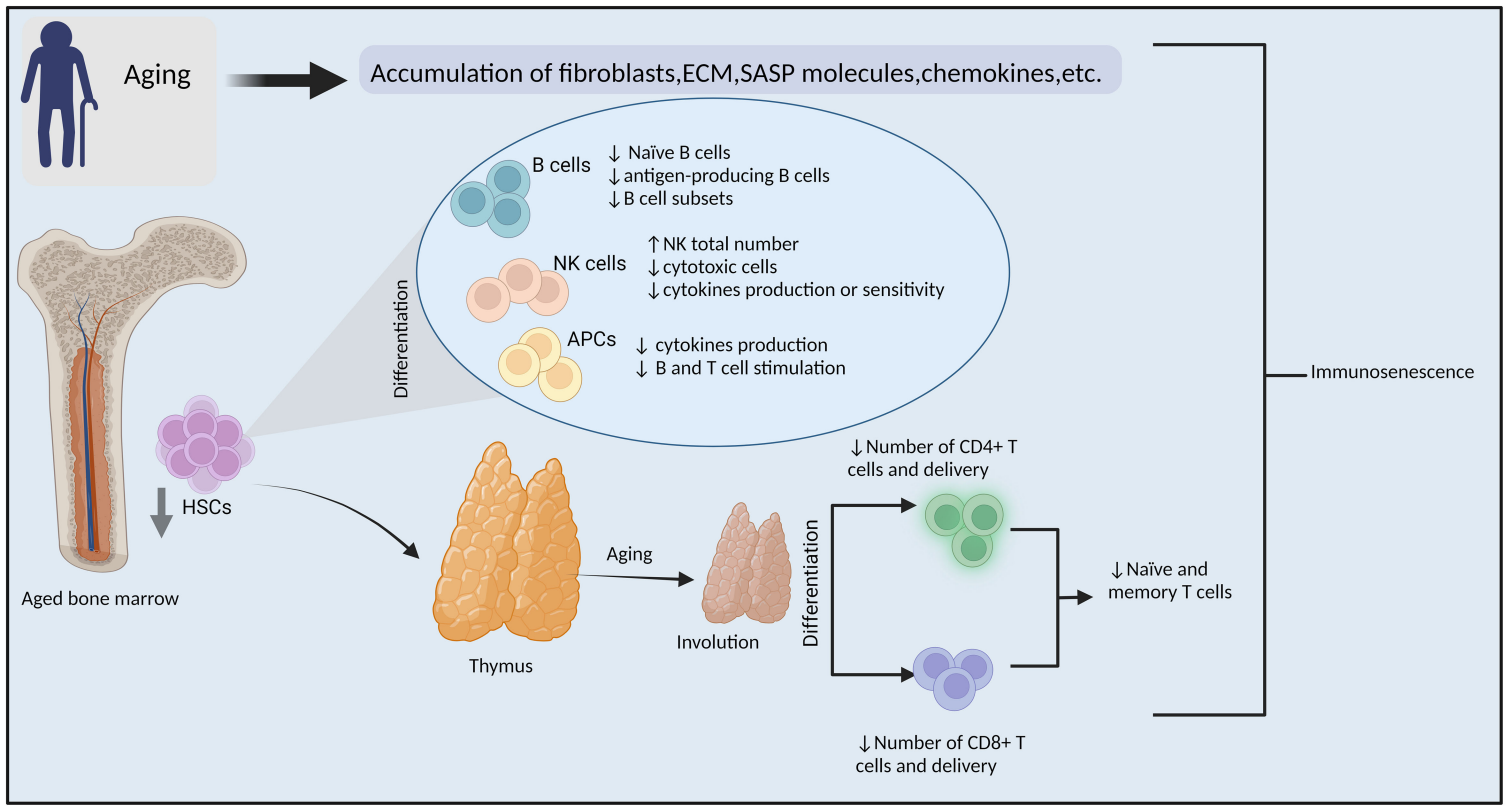
Immunosenescence. Mechanisms of aging-related impairment of the immune system and tumorigenesis. (ECM: extracellular matrix, SASP: senescence-associated secretory phenotype, HSCs: hematopoietic stem cells, NK cells, natural killer cells, APCs: antigen-presenting cells, ↓= Decreased ↑=increased). By BioRender (https://app.biorender.com/).

As life expectancy has increased, geriatric oncology is becoming more critical due to the ever-increasing number of older cancer patients, which has so far concentrated mainly on traditional therapy tolerance (1–3). The correlation between incidence of malignancy and aging has been well established, and age-related immunological deterioration has been acknowledged for even longer (4, 5). It has been demonstrated that age-related accumulation of mutations and DNA methylation contribute to the development of cancer (6–8). Further, recent report have emphasized particular alterations that are contributing to the aging-associated decline of the individual’s immune system (9, 10). Immunosenescence describe the process through which the immune system is assumed to become less capable of performing its functions properly (11, 12). There is growing evidence that the immune response of the elderly to cancer may be diminished or compromised for the following reasons: (1) they have fewer naïve B and T cells, which could leave gaps in their repertoire for neoantigens; (2) their memory T cells, which are capable of recognizing tumor cells, have become exhausted; (3) their immune systems have more immune-suppressive cells (13, 14), and (4) alteration of response of macrophages, and neutrophils with age that are necessary for T cell activation in order to eliminate cancer (15) (**Figure 1**).

**Figure 2:**
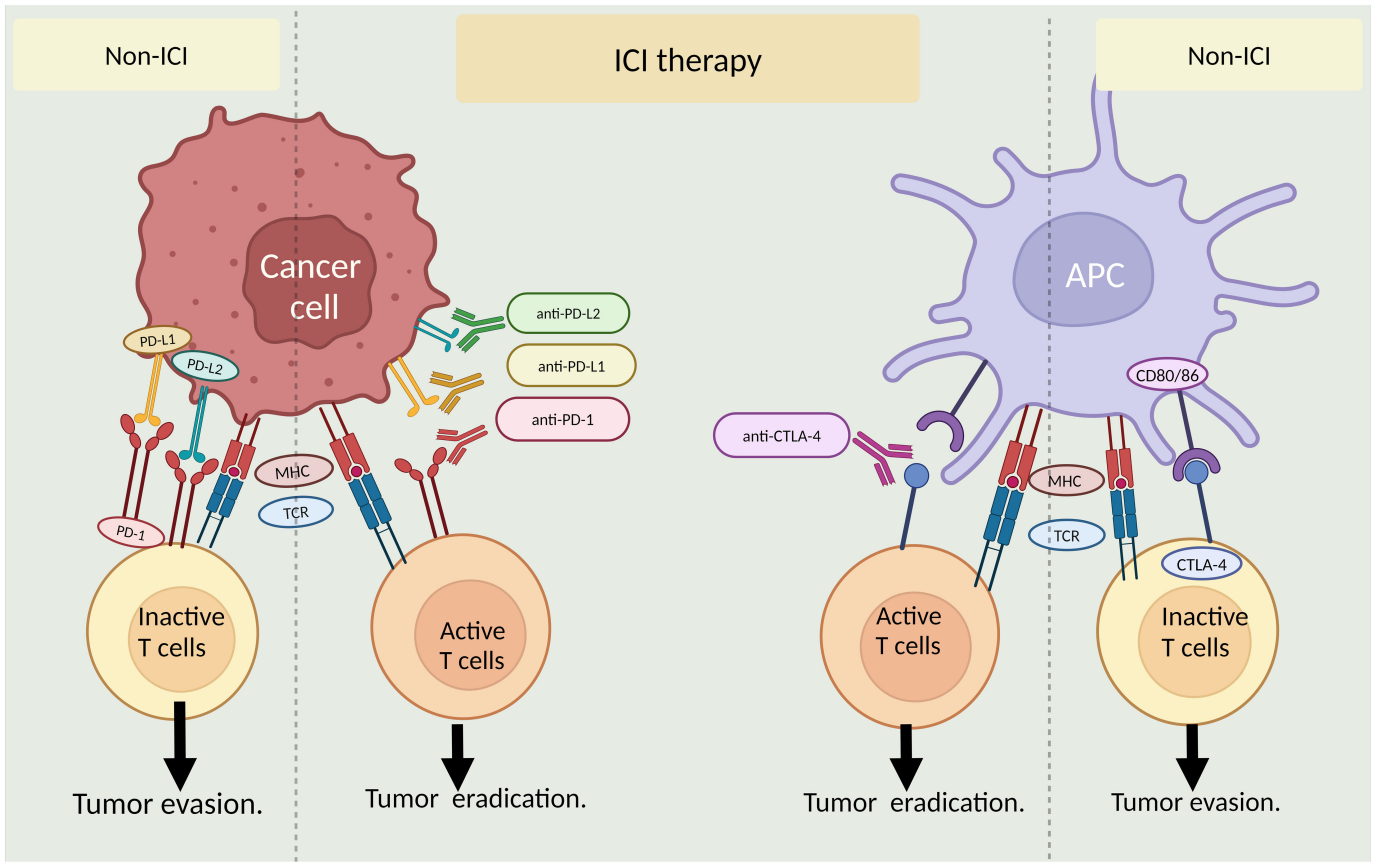
Role of immune checkpoint inhibitors (ICI) in cancer treatment. The left panel shows ICI interaction between cancer cells and T-cells with or without ICI therapy. The right panel illustrates ICI interaction between antigen-presenting cells (APCs)and T-cells in the presence and absence of ICI therapy. MHC: major histocompatibility complex, TCR: T-cell receptor. By BioRender (https://app.biorender.com/).

The past 2 decades have critically advanced our understanding of immune checkpoints. The interaction between T-cell immune checkpoint proteins and their partners on cancer cells (and other cells) transmits "off" signals to T cells upon binding, suppressing the immune response and aiding cancer cell escape. Novel immune-oncology medications known as immune checkpoint inhibitors (ICI) are designed to obstruct this interaction between cancer cells and immune cells, essentially establishing a barrier that thwarts the inhibitory effects. These medications do not stimulate the immune system; rather, they counteract the suppression cancer cells exert on immune cells (**Figure 2**).

Recently, ICI has been applied in many cancer types as a promising treatment option. However, it is essential to comprehend the influence of aging on how the immune system responds to tumors in order to make informed decisions on the creation and implementation of ICI in elderly individuals. With the introduction of groundbreaking antibodies that modulate the immune system to eliminate tumors, immunosenescence has gained attention in oncology (16–19). 

Also critical to the implementation of ICI therapy in an elderly population is the role of immune checkpoint gene expression. The expression levels of some immune checkpoint genes and their corresponding ligands serve as biological markers for different ICI responses. The expression of a widely recognized biomarker, programmed death-ligand 1(PD-L1), shows demonstrated credibility in predicting the patient’s response to anti-programmed death 1 (PD-1)/PD-L1 treatments (20–24). Other biomarkers associated with ICI response are cytotoxic T lymphocyte antigen 4 (CTLA-4), programmed death ligand 2 (PD-L2), cluster of differentiation 80 (CD80), Janus kinase 2 (JAK2), lymphocyte activation gene 3 (LAG-3), hepatitis A virus cellular receptor 2 (HAVCR2), transforming growth factor-B1 (TGF-B1), C-X-C motif chemokine ligand 9 (CXCL9), and cluster of differentiation 86 (CD86) (25–27).

The impact of aging on immune checkpoint gene expression holds considerable significance for the use of ICI. However, the investigation of the correlation between immune regulatory gene expression and aging has not been systematically explored. Age-related alterations in the immune system impact the expression and regulation of significant immune genes, such as PD-L1, as well as the composition and functionality of the tumor-infiltrating lymphocytes (TILs). Elderly individuals may demonstrate distinct PD-L1 expression, diverse degrees of TIL infiltration, and heightened exhaustion of T cells as distinguished by the up-regulation of inhibitory receptors such as PD-1, T-cell immunoglobulin and mucin-domain containing-3 (Tim-3), and LAG-3 (28). These modifications in immune checkpoint genes and TILs that occur with age may have an impact on the effectiveness of ICI and the general response to immunotherapy in elderly individuals (29). Alterations in immune checkpoint receptors, such as CTLA-4 and indoleamine 2,3-dioxygenase (IDO), may also influence the immune response and the efficacy of immunotherapeutic approaches. Although the precise effect of aging on CTLA-4 expression and function remains incompletely understood, research has indicated a rise in CTLA-4 expression in elderly individuals, which may play a role in immune dysregulation (28).

Comprehending the intricate relationship between immune checkpoint genes and age-related immune alterations is imperative in developing tailored and efficacious immunotherapeutic approaches for elderly individuals with cancer. Customizing treatment strategies to consider the distinct immune profiles and features of elderly individuals may augment treatment efficacy within this demographic. Additional investigation is necessary to elucidate the fundamental mechanisms responsible for age-related alterations in immune checkpoint genes. Furthermore, it is imperative to discover new targets that can be utilized to achieve effective immunotherapy in elderly individuals. These pursuits will ultimately enhance the effectiveness and practicality of immunotherapeutic interventions in the context of aging and cancer (**Figure 2**).

## 2 Immune-related genes and aging

### 2.1 Identification of immune biomarkers related to aging

**Figure 3:**
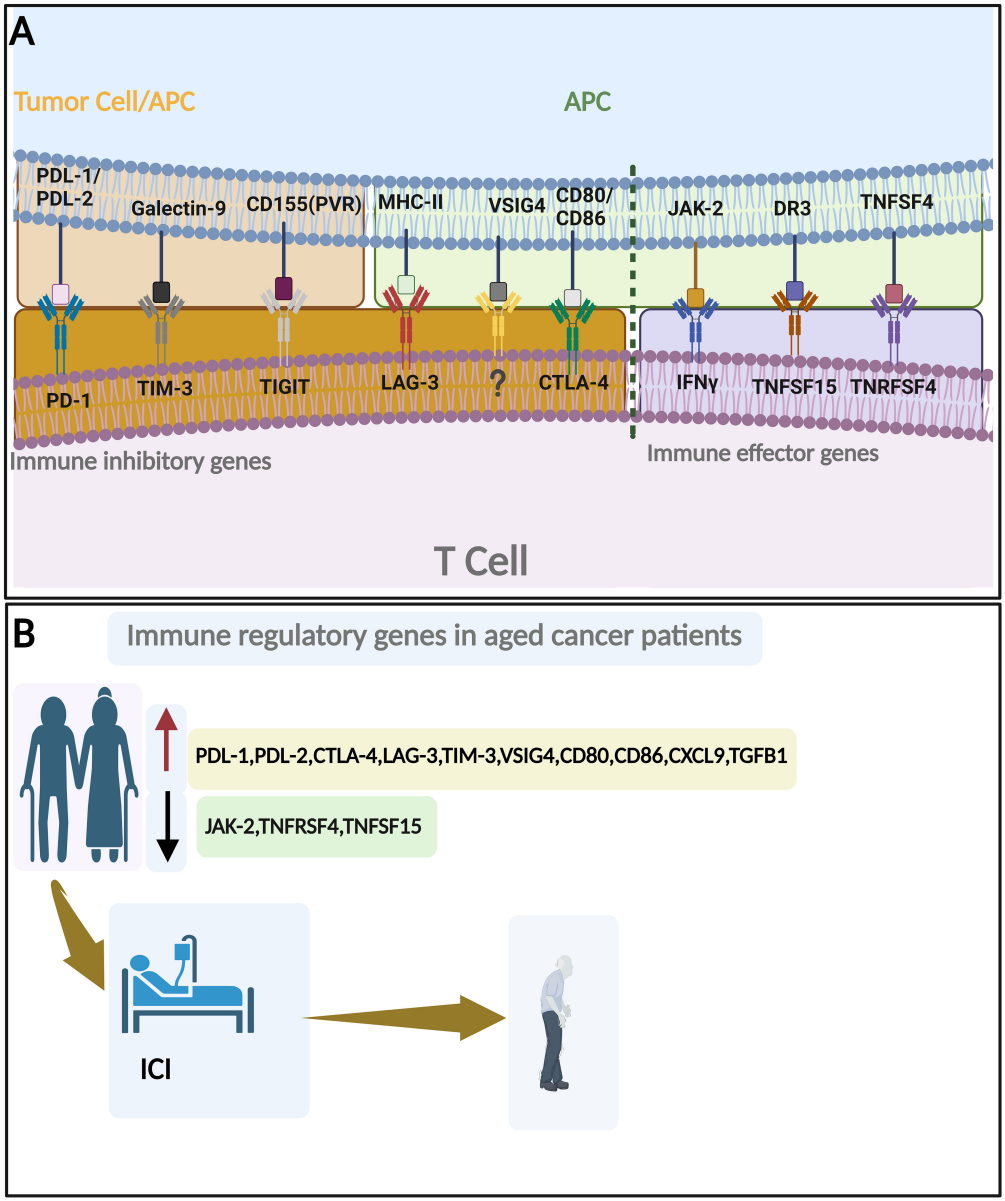
Immune regulatory genes and aging. **(A)** Important immune inhibitory genes (left to the broken line) and immune effector genes (Right to the broken line). **(B)** Change of immune regulatory genes with aging in cancer patients and their roles in ICI response. By BioRender (https://app.biorender.com/).

The aging process plays a crucial role in contributing to and predicting cancer development (30). Aging biomarkers are a combination of biological parameters that hold diagnostic as well as therapeutic values in age-related diseases, including cancer (31). In most cancer tissues, age is linked with changes in somatic mutations, DNA damage repair signatures, and somatic copy-number alterations in most cancer tissues (32–38). Different cancer forms have age-related mutation patterns in well-recognized cancer driver genes. These include mutational signatures linked to APOBEC cytidine deaminase activity, isocitrate dehydrogenase 1 (IDH1), ATRX, POLE/POLD1, PIK3CA, TP53, GATA3, CDH1, ARID1A, KRAS, BRAF V600, CTNNB1, and growth factor signaling pathways (32–35, 39–46). The estrogen receptor-positive (ER+) subtype, associated with a favorable prognosis, is more frequently diagnosed in older individuals. In contrast, the aggressive human epidermal growth factor receptor 2-positive (HER2+) subtype is more prevalent in younger patients (47, 48). Additional genes linked with aging in general include the telomerase reverse transcriptase (TERT), APOE, KL, the FOXO family, SIRT6, the VEGF family, and components of the mitochondrial signaling pathway, chronic inflammation, metabolism, and genes interacting with numerous other pathways such as NF-κB, AMPK, mTOR, P53, and PGC1α (49–57). Finally, among the most altered genes in cancer with age are those of the immune microenvironment, which plays an important role in cancer prognosis and therapy. Hence, identifying and validating immune-related biomarkers associated with aging in cancer holds immense potential for clinical applications (**Figure 3**) (19, 28, 58, 59).

**Figure 4:**
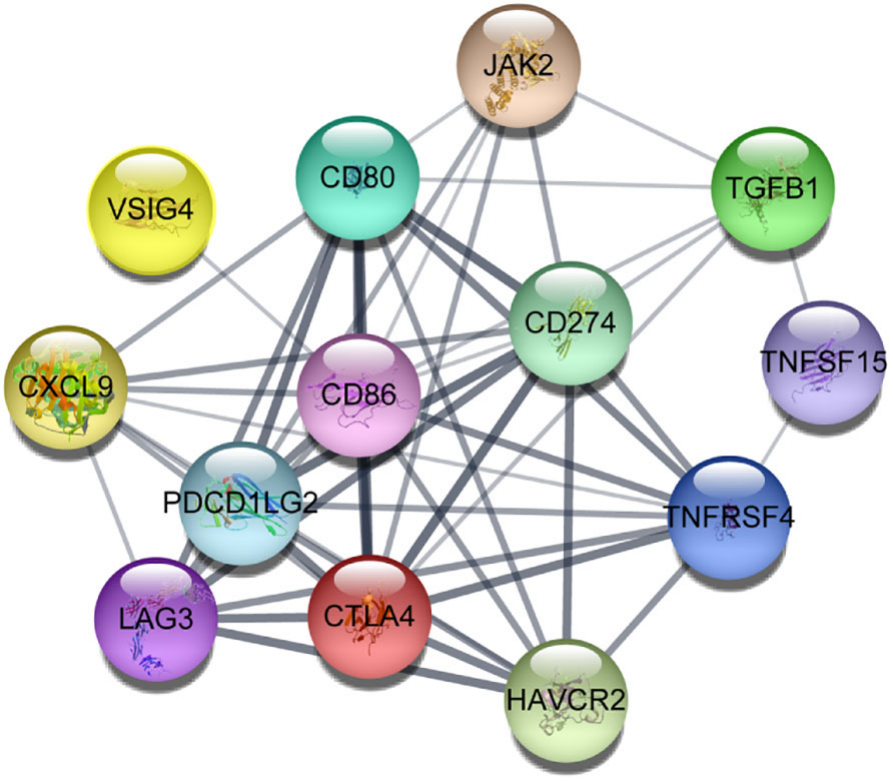
Protein-protein interactions between aging-related critical immune genes in which darker lines represent more robust connections visualized using Cytoscape software.

We constructed a protein-protein interaction network using a set of immunological genes associated with aging. Cytoscape software (60) confirmed that all of these genes had robust interactions with one another (**Figure 4**). Then, we identified the biological pathways and processes underlying this group of genes using the ShinyGO 0.80 web-based tool (61). The associations between these genes and the enrichment of pathways associated with aging and immunology, as documented in the Kyoto Encyclopedia of Genes and Genomes and Gene Ontology, are illustrated in **Supplementary Figure 1** and **Supplementary Table 1**.

These biomarkers may aid in cancer risk stratification, treatment selection, and the development of targeted immunotherapeutic strategies tailored to the unique immune profiles of older individuals. Furthermore, immune-related biomarkers could also serve as endpoints in clinical trials, allowing the assessment of treatment response and the evaluation of interventions aimed at modulating age-related immune dysfunction (62). By unraveling the molecular underpinnings of age-related immune dysfunction and its implications for cancer, researchers strive to improve cancer prevention, diagnosis, and treatment approaches for the growing population of older individuals (**Figure 3**; **Table 1**). In the rest of this section, we will discuss each aging-related immune biomarker in detail.

**Table 1:** Overview of the fluctuations in immune gene expression as a consequence of the aging process.

Immune genes	Full name	Reference	Expression behavior with aging	Findings
PD-L1 (B7 homolog1(B7-H1) or cluster of differentiation 274(CD274)	Programmed death ligand 1	(28, 63–65)	Increased	- PD-L1 expression increases with age in normal as it does in cancer tissue. - Increased PD-L1 expression is increased with aged B cells. - Eradication of PD-L1+ senescent cells by ICI may be a viable anti-aging approach.
CD80(B7-1)	Cluster of differentiation 80	(12, 28, 66, 67)	Increased	- CD80 increased with age in normal and cancer tissues. - CD80 expression differences are a consequence of immunosenescence rather than being influenced by disease or a response to treatment.
HAVCR2 (T-cell immunoglobulin and mucin-domain containing-3 (TIM-3)	Hepatitis A virus cellular receptor 2	(28, 68–70)	Increased	- The expression of HAVCR2 increases with age across diverse tissues in normal and cancer tissues. - Prolonged exposure to cancer-related stimuli alters numerous T cells, prompting an elevation in the expression of co-inhibitory receptor HAVCR2. - Immunosenescence is characterized by higher levels of HAVCR2 in the T cells of elderly patients. - HAVCR2 upregulation renders T cells less responsive and impairs their reactivity.
LAG-3 (CD223)	Lymphocyte activation gene 3	(28, 71, 72)	Increased	- LAG-3 rises with advancing age in normal and cancer tissues. - The PD-1 and LAG-3 co-expression patterns hold significant mechanistic importance, leading to a synergistic reversal of T -cell exhaustion.
PD-L2 (B7-DC, CD273, PDCD1 ligand 2)	Programmed death ligand 2	(28, 65, 73)	Increased	- PD-L2 expression increases with age in normal and cancer tissues. - Both tumor cells and stromal cells demonstrated a higher expression of PD-L2 in the elderly.
TGFB1	Transforming growth factor-B1	(28, 74)	Increased	- TGFB1 increases in cancer and normal tissues as individuals age. - TGF-β elicits senescence in fibroblasts, bronchial epithelial cells, and neoplastic cells.
CXCL9 (Monokine induced by gamma (MIG)	C-X-C motif chemokine ligand 9	(28, 58, 75)	Increased	- CXCL9 rises in cancer as individuals age. - Targeting CXCL9 might mitigate the age-related decline of the vascular system and other physiological systems.
JAK-2	Janus kinase 2	(28, 76)	Decreased	- JAK2 decreases with age in normal tissue. - JAK2 gene is commonly altered in aged blood cells, with the JAK2-V617F mutation being the most prevalent. - Mutations in the JAK1/2 genes may cause resistance to anti-–PD–1 treatment in malignant tissues
TNFRSF4 (ACT35, CD134, OX40, TXGP1L)	Tumor necrosis factor receptor superfamily member 4	(19, 59)	Decreased	-TNFRSF4 was recognized as a gene that is implicated in the process of tumor suppression. - Decrease in the expression of TNFRSF4 in melanoma tissues from older individuals, while its level appears to be elevated in the younger age group.
TNFSF15 (TNF Superfamily Ligand TL1A, Vascular endothelial cell growth inhibitor)	Tumor necrosis factor (ligand) superfamily, member 15	(59)	Decreased	-TNFSF15 was identified as a gene linked with tumor suppression -Expression was higher in younger ccRCC clear cell renal carcinoma tissues than in older patients.

### 2.2 PD-L1

PD-L1, also known as CD274, is a cell surface protein that plays a significant role in immune response regulation by binding to T cells through PD-1, thereby suppressing cancer immunity, and serving as a signal to evade detection (77, 78). The PD-1 interacted with PD-L1 on the surface of T-cells dephosphorylates T-cell receptors, specifically involving SHP-1/2. The observed effect of this phenomenon is the inhibition of T-cell mediated cytotoxicity toward malignant cells, which is attributed to the reduction of T-cell proliferation and activity (77, 79). ICI, specifically monoclonal antibodies targeting PD-1 and/or PD-L1, have been employed in various cancer therapies (80). Despite the considerable clinical advantages of PD-1/PD-L1 blockade therapy for various cancer types, the response rates of patients remain below 40%, and the underlying mechanism is not entirely understood (77). Still, distinct types of cancers exhibit high PD-L1 levels that facilitate immune evasion by cancer cells (81).

The intricate modulation of PD-L1 expression in malignant cells encompasses a multifaceted interplay of intricate signaling cascades, including but not limited to nuclear factor-κB (NF-κB), mitogen-activated protein kinase (MAPK), molecular target of rapamycin (mTOR) signal transducer and activator of transcription (STAT), and cellular myelocytomatosis (c-myc). Additionally, the degradation of PD-L1 protein occurs by many pathways, including proteasomes and lysosomes. These mechanisms contribute to the enhanced efficacy of cancer immunotherapy but may be limited by changes related to aging (77, 82). Multiple studies have reported a higher level of PD-L1 expression within the geriatric population compared to the younger cohort (28, 63, 64). It has been observed that senescent cells increase the expression of PD-L1, thereby leading to the deactivation of immune cells. The upregulation of PD-L1 during senescence is contingent upon the activation of the proinflammatory pathway (63). In addition, the factors secreted by cells undergoing senescence can induce an increase in PD-L1 expression in non-senescent cells, which occurs through the JAK-STAT signaling pathway. Furthermore, the research (63) indicates that the intervention of prolongevity through rapamycin reduces PD-L1 expression in aging cells. Finally, it was discovered that PD-L1 expression is increased in multiple tissues in mice that have undergone natural aging, as well as in the lungs of patients with idiopathic pulmonary fibrosis (63). Collectively, the findings indicate that the process of senescence and aging is linked to an increase in the expression of PD-L1. Hence, the targeting of PD-L1 presents a promising avenue for developing new therapeutic interventions aimed at addressing the pathophysiological processes linked to senescence and age-related disorders, including malignancies (63).

The process of surveillance by the immune system, which involves the recognition and elimination of aberrant cells, for instance, cancer cells, is subject to negative regulation by immunological checkpoints, including those associated with senescent cells. In order to investigate this inquiry, scholars conducted an analysis of the manifestation of numerous immune-regulated checkpoint molecules in human fibroblast cell line HCA2, which is recognized as an in vitro representation of senescent fibroblasts. The overall concentration of PD-L1 was shown to be dramatically and particularly elevated in nutlin3a-induced senescent cells(n-Sen) and DNA damage-induced senescent cells(d-Sen) when compared to starvation-induced quiescent cells (64, 83, 84). The findings additionally demonstrated that senescent cells exhibit heterogeneous in the expression of the immunological checkpoint protein PD-L1. Furthermore, it is seen that there was an age-related accumulation of PD-L1+ senescent cells in vivo. Cells expressing PD-L1 are resistant to T cell surveillance, even when a senescence-associated secretory phenotypes (SASP) is present, whereas cells not expressing PD-L1 are vulnerable to T cell surveillance. “P16 positive cells” are those containing observable amounts of the p16 protein, a key player in regulating the cell cycle and frequently associated with cancer. Moreover, p16 is among the regulatory factors engaged in initiating and sustaining cellular senescence (85).

The in vivo examination of senescent cells (namely, p16+ cells) using single-cell analysis demonstrated a positive association between PD-L1 and elevated levels of the SASP. In accordance, the administration of PD-1 antibody to mice that are naturally aging, as well as to a mouse model with normal livers or induced nonalcoholic steatohepatitis, results in a decrease in the overall quantity of p16+ cells in vivo, as well as a reduction in the PD-L1+ population in an activated CD8+ T cell–dependent manner (85). Thus, this intervention effectively improved several aging-related characteristics The findings of this study indicate that the varied expression of PD-L1 plays a crucial role in the buildup of senescent cells and inflammation linked to the aging process. Furthermore, the removal of PD-L1+ senescent cells by immune checkpoint inhibition shows promise as a potential approach for anti-aging treatment (64, 83). Recently, researchers found that PD-L1 expression of CD8+ effector T cells was significantly higher in aged mice than younger mice and anti-PD-L1 immunotherapy led to a decrease in cell proliferation in vitro and a reduction in anti-tumor immune response in older hosts, as compared to their effects in young mouse lymphoma models (86, 87). A separate investigation observed that the levels of PD-L1 and IDO1 were elevated in the brains of healthy adult humans as they aged. Additionally, the aging process was associated with increased circulating regulatory T cell numbers and decreased CD8+ T cells (88). While all of the evidence suggests that cancer patients with high PD-L1 expression benefit more from ICI, whether older patients with cancer who express PD-L1 will respond better to ICI is not completely known. Patients over 65 or even 70 are sometimes ineligible to participate in various ICI investigations; this group is generally too small to evaluate. However, because people are frequently diagnosed with cancer around the age of 65, studies on the efficacy and safety of ICI in older patients are required (28, 58, 89).

### 2.3 CD80

The cluster of differentiation 80 (CD80), also known as B7-1, is a cell surface protein expressed on tumor cells or APCs (90). It interacts with both con-inhibitory CTLA-4 and co-stimulatory CD28 receptors, thereby playing a vital function in immune response regulation (91–93). The competitive binding of CD28 and CTLA-4 to CD80 is subject to regulation by various factors, including affinity to CD80 and the expression kinetics of CD28 and CTLA-4 in T cells (93). The affinity of CTLA-4 to CD80 is approximately ten times greater than its affinity to CD28. Also, CTLA-4 is predominantly expressed on T cells that have been activated, whereas CD28 is expressed on T cells in a constitutive manner (94). Prior studies have indicated that the expression level of CD80 might influence the pro-/anti-oncogenic function of CD80 on neoplastic cells (93–97). The downregulation of CD80 expression is a mechanism tumors employ to evade immune surveillance because CTLA-4 exhibits a higher affinity for CD80 than CD28, does resulting in preferential binding to the CTLA-4. Conversely, the upregulation of CD80 induces T-cell activation and enhances tumor rejection. Moreover, research has demonstrated that a soluble variant of CD80 has the capability to attach to PD-L1, thereby impeding the PD-1/PD-L1 axis (98). Furthermore, the interaction between PD-L1 and CD80 in a cis configuration, rather than a trans configuration, hinders the immunosuppressive axis of PD-1/PD-L1 and CTLA-4/CD80 (99, 100). Antibodies that block PD-L1 prevent the interaction between CD80 and PD-L1 on dendritic cells that are associated with tumors, thereby promoting the anti-tumor immune response mediated by CD80 (93, 101). The expression behavior of CD80 appears altered with aging. Furthermore, it has been observed that the expression levels of immune checkpoint molecules, including Tim-3, T cell immunoreceptor with immunoglobulin and ITIM domain (TIGIT), and CTLA-4, exhibit a rising pattern with advancing age (12). Studies investigating the relationship between aging and CD80 expression in the era of ICI have demonstrated a positive correlation between advanced age and elevated CD80 levels (28, 58). A group of researchers found that the population of CD14+ monocytes remained stable; however, there was an age-related increase in the expression of major histocompatibility complex (MHC) II cell surface receptor (HLA-DR), CD80, and CD86 by monocytes. Nevertheless, no discernible distinctions were observed upon juxtaposing Alzheimer disease (AD) patients with age-matched healthy controls or subsequent to administering rivastigmine treatment to AD patients (67). The findings suggest that alterations in the manifestation of HLA-DR, CD80, and CD86 are attributable to immunosenescence rather than AD pathology or the administration of rivastigmine to AD patients. A recent investigation demonstrated that in the context of aging, neurodegenerative disease, central nervous system inflammation, and injury, antigen-presenting cells, such as microglia or infiltrating macrophages, increase the expression of immunomodulatory proteins, such as CD80 (67, 102). A universal deficiency in the enhancement of the co-stimulatory molecule CD80 on monocytes in relation to age was noted across all TLR ligands that were examined, including TLR1/2, TLR2/6, TLR4, TLR5, and TLR8 (66, 103). The profound connection between the upregulation of CD80 expression upon TLR engagement and the development of a protective antibody response to influenza vaccination has significant implications for adaptive immunity. This observation diverges from the trends observed in other findings, highlighting the nuanced role of CD80 and the complexity of its regulation in the aging process and cancer. Although the CD80-encoded protein plays a role in tumorigenesis and progression, especially when interacting with CTLA-4, there is evidence to suggest that patients with elevated CD80 expression may experience advantages when treated with ICI-like antibodies targeting CTLA4 or PD-1/PD-L1 (104). However, the response to ICI treatment in older cancer patients expressing CD80 remains dichotomous and requires additional research to elucidate. 

### 2.4 HAVCR2

The T-cell immunoglobulin and mucin domain 3(TIM-3) protein, which is encoded by the HAVCR2 gene, is a cell surface molecule containing both immunoglobulin and mucin domains. Its initial identification was as a cell surface marker that exhibits specificity towards interferon (IFN-γ)-producing CD4+ T helper 1(Th1) and CD8+ cytotoxic 1(Tc1) cells (105). Tim-3 is a member of the Tim gene family, which is located in syntenic chromosomal regions in humans (5q33.2) and mice (11B1.1) that have been linked to allergies and autoimmune diseases (106, 107). The research conducted by Monney et al. provided initial evidence of Tim-3's inhibitory role as a checkpoint receptor on T cells by introducing Tim-3 monoclonal antibodies in vivo, which worsened symptoms in an experimental autoimmune encephalomyelitis model (108). Subsequent to the aforementioned events, two separate investigations concluded that interfering with the interactions between Tim-3 and its ligand, using Tim-3-Ig or Tim-3 mAb, led to increased Th1 responses and promoted the development of autoimmune diabetes in nonobese diabetic mice (109, 110). Despite these findings, the lack of a classical inhibitory signaling motif in the cytoplasmic tail of Tim-3 cast doubt on the protein’s inhibitory function. The association between germline loss of function mutations in HAVCR2 and two diseases resulting from hyperactivated T and myeloid cells, hemophagocytic lymphohistocytosis (HLH) and subcutaneous panniculitis-like-T-cell(SPTCL), establish Tim-3 as a negative regulator or “immune checkpoint” (111, 112). Tim-3 expression is simultaneously controlled and expressed on CD4+ and CD8+ T cells in conjunction with other immune checkpoint receptors, including PD-1, LAG-3, and TIGIT (113, 114). In general, with aging, Tim-3 demonstrates increased expression. Senescent T cells have been found to possess atypical characteristics, including reduced expression of CD27 and CD28 and increased expression of CD57, killer cell lectin-like receptor subfamily G, Tim-3, TIGIT, and cytotoxic T-lymphocyte-associated protein 4. These molecular alterations have been linked to the formation of malignant tumors (12, 115, 116). Certain researchers contend that the dysfunction of T-cells with aging is distinct from the exhaustion of T-cells, which is a condition of reduced cell response that occurs as a result of prolonged exposure to situations such as viral infection and cancers (69). Continuous antigen activation leads to T cell depletion and a steady increase in the levels of inhibitory checkpoint receptors [such as PD-1, CTLA-4, LAG-3, inducible co-stimulator (ICOS), Tim-3, and killer cell lectin-like receptor G1(KLRG-1)] on CD4+ T cell that consequently downregulate T cell receptor (TCR)-induced intracellular signaling (117, 118). Tim-3 expression uniquely identifies the most dysfunctional or terminally exhausted subset of CD8+ T cells in malignancies (119, 120). Targeting of Tim-3 is a potential therapeutic opportunity. Co-blockade of the Tim-3 and PD-1 pathways has demonstrated exceptional efficacy in preclinical cancer models for both solid and hematologic tumors (121, 122). Current clinical trials investigate anti-Tim-3 in conjunction with anti-PD-1 for treating solid malignancies due to their persistent antigen T-cell stimulation (120, 123). Considered a detrimental regulatory factor of T-cell death, TIM-3 is expressed on the Th1 cells' surface and achieves its function by binding to its ligand, galectin-9 (124). The inhibitory effects of TIM-3 extend to innate and adaptive immunological reactions, particularly those involving CD8+ and Th17 cells, as well as tolerant acquisition mediated by regulatory T cells (Tregs) (105, 125). HAVCR2 expression was higher in most tumors, including glioblastoma and low-grade glioma (GBMLGG), lung adenocarcinoma (LUAD), prostatic adenocarcinoma (PRAD), and sarcoma (SARC) (70). It has also been shown that age leads to an increase in Tim-3+ murine T cells, which in turn decreases T cell’s proliferative capacity (126, 127). A prior study has indicated that overexpression of immune exhaustion markers like TIM-3 can result in resistance to ICI therapy. Consequently, targeting this exhaustion marker on its own or in conjunction with PD-1 may lead to more favorable clinical responses (128). Despite TIM-3 heightened expression in the elderly population, the impact of this expression on ICI response in older cancer patients warrants further investigation (28).

### 2.5 LAG-3

The LAG-3 protein is classified as a type I transmembrane cell-surface protein that operates in the immune system as an inhibitory co-receptor, analogous to the PD-1 and CTLA-4 proteins (129). The identification of the LAG-3 encoding gene dates back to 1990 when complementary DNA clones expressed in an IL-2-dependent CD3-negative natural killer-cell line were analyzed (130). Researchers observed that the LAG3 gene encodes a membrane protein of four extracellular domains belonging to the immunoglobulin superfamily (131). It has been shown that exhausted CD4+ T lymphocytes have an increased copy number of the LAG3 gene, which subsequently downregulates the TCR-induced intracellular signaling (69, 118). Comparably, LAG-3 upregulation is also observed in dysfunctional immunosenescent cells (69, 118, 127, 132). Recent research has shown that as individuals age, there is a drop in the production of the proapoptotic protein Bim, which leads to an increase in the lifetime of naïve CD4+ T cells. This finding has led to the notion that the extended lifespan of these cells makes them more susceptible to accumulating defects (72). Additionally, naïve T cells have the potential to develop a’semi-memory’ phenotype as they mature, signifying their entry into differentiation pathways. In turn, aged naïve T cells have shown expression of molecules typically associated with prolonged stimulation and exhaustion, including PD-1 and LAG-3 (71). T-cell exhaustion can result in a reduction of cytokine production that is responsible for tumor-killing (132). Hence, elevated levels of LAG-3 restrict the activation of T-cells, so diminishing their capacity to target and eliminate cancerous cells. Conversely, suppression of LAG-3 reestablishes the normal functioning of T-cells (133).

LAG-3 shows potential as a therapeutic target in cancer. Recent work has demonstrated a significant association between the expression of LAG-3 and the presence of LAG-3-positive cells within tumors, and adverse clinical outcomes, tumor advancement, and unfavorable prognosis across various cancer types such as lymphomas, renal cell carcinoma, colorectal cancer, breast cancer, and head and neck squamous cell carcinoma (HNSCC) (134). The investigators’ initial focus was to examine the effects of LAG-3 inhibition as a standalone treatment; however, it was shown that employing a single-agent approach yielded minimal effectiveness. In contrast, the simultaneous inhibition of LAG-3 and PD-1 receptors had a synergistic effect, leading to a reduction in tumor growth and the enhancement of anti-tumor immune responses, which may be attributable to differences in the inhibitory mechanisms and or expression patterns of PD-1 and LAG-3 (129). Moreover, the protein expression levels of LAG-3, PD-1, and TIM-3 in NSCLC tissue were evaluated in a previous study that concluded a negative association between LAG-3 and anti PD-1 therapy response (135). In March 2022, the FDA approved the combined use of anti–PD-1 antibody nivolumab and the LAG-3 blocker relatlimab-rmbw (Opdualag) for the therapy of individuals aged 12 and above who have unresectable or advanced melanoma (136, 137). Importantly, a potential factor in resistance to anti–PD-1/anti–PD-L1 immunotherapies in cancer is LAG-3 and PD-1 co-expression, which may lead to significant T-cell dysfunctionality (138). In accordance with this, PD-1 and LAG-3 co-blockades increase many T-cell anti-tumor activities (138). In a nutshell, LAG-3 has emerged as a viable option for cancer immunotherapy, but more study of these strategies is critical, particularly in the older population, where LAG-3 expression is significantly higher than in younger people (28, 58, 139).

### 2.6 PD-L2

PD-L2, which is alternatively referred to as B7-DC or CD273, is categorized within the B7 family members. This protein’s extracellular region comprises an immunoglobulin (Ig)-like V-type domain and an Ig-like C2-type domain; it is classified as a type I transmembrane protein. The expression of PD-L2 has been observed in both neoplastic and immunological cells, and its elevated expression has been established to be a significant factor in both tumor formation and evasion of immune surveillance (140). PD-L2 overexpression was seen to correlate with a negative prognosis in individuals diagnosed with HNSCC, adenoid cystic carcinoma (ACC), esophageal cancer, and other related conditions (141). Moreover, a study conducted on colon cancer revealed that the expression of PD-L2 exhibited a positive correlation with neuroinvasion and a negative correlation with CD8+ tumor-infiltrating lymphocyte density (142). PD-L2 has been identified as a significant independent prognostic factor in the context of anti-PD-1 immunotherapy. The proposition put forth by researchers is that PD-L2 has a significant function in circumventing anti-tumor immunity, similar to PD-L1. This implies that for effective immunotherapy in cancers that express PD-L2, such as HNSCC, RCC, and LUSC, it is imperative to take into account the blockade of PD-1/PD-L2 (141).

In one investigation, the tumors of aged mice exhibited a greater abundance of PD-L2+ B16 tumor and stromal cells, as well as a higher PD-L2 mean fluorescence intensity, compared to tumors of young mice (65). Variations in the expression of PD-L2 and PD-1 may shed light on the more effective response of aged mice to PD-1 inhibitors compared to PD-L1 inhibitors. This enhanced efficacy can be attributed to the ability of anti-PD-1 therapies to block the inhibitory signals originating from both PD-L2 and PD-L1 simultaneously. This dual blockade potentially offers a more comprehensive therapeutic approach, especially in contexts where both these ligands are expressed. Prior research has indicated that PD-L2 levels in B cells rise with the aging of individuals in many organs, including blood, bone marrow, and spleen. Furthermore, aged PD-L2+ B cells modulate CD4+ T-cell activity differently from their younger counterparts, and this modulation depends on PD-L2. Additionally, PD-L2+ B cells impede the growth of subcutaneous MC38 colon cancer. Similarly, age was significantly associated with PD-L2 level (73).

It is worth noting that both the overproduction and inadequate removal of senescent cells could potentially play a role in the development of pathological aging. Efforts are underway to ascertain the mechanisms by which senescent cells evade immune effector-mediated destruction. These mechanisms entail the expression of immunosuppressive molecules, such as PD-L1 and PD-L2, on the surface of cells to bind to PD-1 receptors in T cells, and tolerogenic variants of MHC class-I are also involved. Furthermore, senescent cells can release certain substances that can potentially attract immunosuppressive and pro-inflammatory cells toward the surrounding microenvironment (143). Therapeutic intervention could be targeted towards each immune evasion mechanism, such as obstructing the interaction between PD-1 and PD-L1 or PD-L2, enhancing the expression of immunogenic MHC class-I molecules, and eradicating immunosuppressive cell populations. The study conducted by Chaib et al. revealed a noteworthy finding wherein the expression of PD-L2 was observed to be heightened in various cellular models of senescence, while PD-L1 expression remained unaffected. Notably, eradicating tumors induced by doxorubicin treatment can be achieved through anti PD-L2 treatment by decreasing the presence of senescent cells within the tumor and limiting the recruitment of immunosuppressive cells (144). The suggestion is that PD-L2, much like PD-L1, plays a vital function in evading the body’s anti-tumor immune mechanisms. This implies that to achieve the most effective immunotherapy in PD-L2 expressing cancers like HNSCC, RCC, and LUSC, it’s essential to consider blocking the PD-1/PD-L2 interaction (141). However, our understanding of the regulatory mechanisms governing PD-L2 remains somewhat unclear, and as of now, there are no established clinical practices or trials for immunotherapy strategies targeting PD-L2. This knowledge gap is especially pronounced when it comes to elderly patients who have high PD-L2 expression, making it imperative for further in-depth research in both areas (58, 68).

### 2.7 TGFB1

Transforming growth factor-β (TGF-β) is a group of related proteins produced by all cells and functions through a complex cell surface receptor system. Since its identification as a secreted factor that triggers reversible phenotypic changes in specific fibroblast cell lines, TGF-β has been the subject of extensive research regarding its involvement in malignancy development and progression (145). Three groups of findings (145, 146) propelled the development of these studies: (1) Tumor cell lines and tissues frequently exhibit elevated levels of TGF-β expression in comparison to healthy cells or tissue; (2) The growth inhibitory effect of TGF-β observed in normal epithelial cells is frequently compromised in carcinomas; and (3) The autocrine and paracrine TGF-β signaling pathways in tumor cells regulate significant alterations in gene expression, which suppress the epithelial phenotype, facilitate invasion and dissemination, foster stem cell characteristics, release immunosuppressive cytokines, and contribute to cancer drug resistance (145, 146). In order to achieve sustained remission, therapeutic strategies that target TGF-β signaling ought to concentrate on cancer cells as well as immunological and stromal components of the tumor microenvironment (TME) (146). Increased TGF-β signaling has been correlated with resistance to many anti-cancer treatments, including molecularly targeted medicines and chemotherapy as well as immunotherapies (147). Consequently, only a subset of individuals among those with diminished TGF-β signaling patterns exhibit a positive response to anti-PD-1 or -PD-L1, anti-CTLA-4, and CART cell therapies. The potential benefits of therapeutic repression of TGF-β signaling to improve response to anti-PD-1/-PD-L1 are expected to be substantial. Unlike chemotherapy, immunotherapy, including TGF-β blockade, can yield delayed or unpredictable responses partly due to the cascade of immune events required for a productive anti-tumor cytotoxic response (148–152).

Although the effect of TGF-β signaling varies significantly depending on the specific cell kind and cellular environment, several growth-inhibiting processes are thought to play a role in the emergence of senescent characteristics. According to a multitude of findings (74) pertaining to pathology associated with aging, the influence of TGF-β signaling can be broadly categorized into two dimensions: (1) the compromise of TGF-β signaling in specific cellular populations, as evidenced by the decline in neuroprotective functions in AD and the attenuation of TGF-β-mediated suppression of growth in tumor, and (2) the persistent rise of TGF-β signaling that is associated with tissue fibrosis, continual inflammation, diminished regenerative capability, and metabolic dysfunction reported in AD, wasting of muscles, weight gain, and various other pathological conditions. Alterations in TGFB1 influenced by the aging process affect Smad2/3 interactions, notably with transcriptional intermediary factor 1 (Tif1γ), and shift the activin receptor-like kinase (ALK)1/ALK5 ratio, favoring Smad 1/5/8 pathways (74, 153–155). This change underlies aging-associated dysregulation in processes like metabolism and angiogenesis, contributing to cancer progression where the TGF-β plays a pivotal role. TGF-β's role in inducing senescence, affecting the tumor microenvironment (TME), and modulating PD-L1 expression highlights its impact on the efficacy of ICI therapy (156, 157). A plethora of studies have highlighted the numerous regulatory mechanisms behind the context-dependent influence of TGF-β signaling in the past few years (158). Nonetheless, the intricate relationship between TGFB1 signaling and aging remains enigmatic within various pathological contexts. Further investigations are necessary to assess the response to ICI therapy in elderly cancer patients characterized by elevated levels of TGFB1 (28).

### 2.8 CXCL9

CXCL9 is a member of the ELR-negative CXC chemokine subfamily that is activated by interferon-γ (IFN-γ) and released by tumor-associated dendritic cells (159). The chemokine CXCL9 facilitates the infiltration of lymphocytes with tumor-suppressive properties into solid tumors by activating its receptor CXCR3 (160). In preclinical cancer models, CXCR3 chemokine activity was shown to be crucial for effective immune checkpoint inhibition, owing to CXCR3's role in both T-cell recruitment and T-cell activation (161–163). A recent report on over 1000 patients with various tumor types indicated that CXCL9 gene expression and tumor mutational burden are highly effective markers for predicting the response to ICI (164). CXCR3 serves as the co-receptor for CXCL9, CXCL10, and CXCL11. Within the TME, the CXCL9/10/11-CXCR3 signaling pathway has the potential to elicit anti-tumor immunity through various mechanisms. These mechanisms include facilitating the chemotactic migration of CXCR3-activated immune cells to tumor sites and activating the STAT and phosphoinositide 3-kinase (PI3K)-Akt signaling pathways. This activation results in the upregulation of PD-L1 expression, which is typically indicative of a favorable response to ICI (165, 166). Elevated levels of CXCL9 and CXCL10 have been observed to be associated with heightened tumor CD8+ T cell density and enhanced patient survival across various cancer types (58, 167, 168).

In the context of aging other than cancer cells, research has identified that the most significant factor influencing the inflammatory aging clock (iAge)was the chemokine CXCL9, which has been implicated in the process of cardiac aging, unfavorable cardiac remodeling, and impaired vascular function (75). In that study, the elderly group exhibited elevated expression levels of immune genes linked to chemokines, specifically CCL5, CXCL9, and CXCL10. In addition, it has been observed that aging endothelial cells in both humans and mice exhibited a decline in functionality, cellular senescence, and characteristic phenotypes of arterial stiffness. Notably, these effects could be mitigated through the inhibition of CXCL9. Ultimately, the authors ascertain a significant function of CXCL9 in chronic inflammation associated with aging and established a multimorbidity measure that can promptly detect age-related clinical phenotypes (75). Indeed, Furman and his colleagues have recently published a study demonstrating that iAge exhibits a correlation with various diseases and remarkable longevity. By employing deep learning techniques in examining the blood immunome of a cohort comprising 1,001 individuals, a modifiable chemokine CXCL9 was identified as being linked to cardiac aging. This discovery holds promise for the early detection of age-related pathology and presents a potential target for therapeutic interventions. In light of research has predominantly indicated that CXCL9 is linked to the response to ICI in advanced cancer patients, the observed increase in CXCL9 expression in older individuals, as a response marker for emerging immune-oncology treatments in this population (28, 58, 169).

### 2.9 JAK2

Janus kinase 2 (JAK2) is a cytoplasmic non-receptor tyrosine kinase that plays aa vital role in the JAK/STAT signaling pathway, a crucial regulator of, differentiation, survival, and immune response. Indeed, previous studies unveiled that the classical inflammatory factor JAK2 was associated with PD-L1 (170, 171). Somatic mutations that occur with advancing age in hematopoietic stem cells result in clonal sub-populations forming in the bloodstream of elderly individuals. This phenomenon is commonly linked to chronic inflammatory alterations, cardiovascular disease, and malignant progression. JAK2 is one of the genes that frequently undergo mutations during the aging process. In line with the conditions associated with these clonal modifications, the aberrant activation of JAK2 in myeloproliferative neoplasms is associated with the excessive proliferation of myeloid precursor cells, the aberrant release of inflammatory cytokines, and an increase in agglutination and thrombosis (76). The JAK2 p.V617F mutation is frequently detected in myeloproliferative neoplasms and has been associated with various complications (172).

Researchers have revealed that resistance to T-cell based therapies across both in vitro and in vivo models of breast cancer is predominantly due to the disruption of JAK2, a kinase integral to the interferon-gamma signaling pathway. This discovery highlights a new resistance mechanism to such therapies and suggests that monitoring JAK2 and interferon-gamma responses could effectively indicate patient response to immunotherapy treatments (173). Loss-of-function mutations in the JAK1/2 genes may cause resistance to anti-PD-1 treatment in certain malignant tissues (174). This instance emphasizes the potential importance of amplifying the PD-L1, PD-L2, and JAK2 gene clusters as a biomarker for sensitivity to chemotherapy (175). Significantly, the enhancement of this group of genes seems to make individuals vulnerable to nivolumab, irrespective of the existence of rearranged during transfection (RET) rearrangement, a well-known mutation that stimulates non-small cell lung cancer (NSCLC). This prompts the inquiry of whether including regular screening for rare but powerful markers of ICI sensitivity might aid in choosing the first systemic treatment for patients with NSCLC (175). The tumor cell membrane has a transmembrane-attached peptide-MHC class I receptor maintained by beta-2-microglobulin(B2M). Additionally, it contains the PD-L1 ligand and IFNGR1/IFNGR2 heterodimer, which are bound by JAK1 and JAK2 (176). The activation of phospho-STAT downstream of JAK1/JAK2 is known to facilitates the regulation of various targets, including MHC class I and PD-L1 expression, through its interaction with tumor cell DNA (176). According to a previous study, the activation of the oncogenic JAK2 gene increases the expression of PD-L1 on numerous cell types, including monocytes, megakaryocytes, MDSCs, and platelets. This upregulation is mediated by the JAK2-STAT3 and JAK2-STAT5 signaling pathways, which elicited a substantial response to ICI therapy (177). Overall, the role of the JAK2 gene in cancer treatment highlights its potential as a target, while its relevance to older cancer patients requires further investigation (28).

### 2.10 TNFRSF4

Tumor necrosis factor receptor superfamily member 4 (TNFRSF4), also known as CD134 or OX40, is a cell surface receptor protein that belongs to the tumor necrosis factor receptor superfamily and is predominantly expressed on activated T cells (178). Previous studies showed that TNFRSF4 facilitates the activation of the NF-kappa-B pathway by mediating tumor necrosis factor receptor-associated factor 2 (TRAF2) and TRAF5 (179, 180). The downstream signaling pathways of TNFRSF4 have also been identified to include the NFAT and P13K/PKB pathways (181, 182). The principal role of TNFRSF4 is to enhance the replication, growth, and survival of T cells as well as the production of cytokines through promoting the activity of the abovementioned signaling processes (179). The TNFRSF4 molecule has been identified as a co-stimulatory agent that may significantly promote effective antitumoral responses in sarcoma, melanoma, and breast cancer, as suggested by preclinical research (183, 184). The available data indicate that the amplification of anti-PD-1 therapy can be enhanced by targeting TNFRSF4, owing to the ability of TNFRSF4 agonism to amplify the expression of PD-L1 (185). Furthermore, tumor cytotoxicity can be augmented by upregulating TNFRSF4 within chimeric antigen receptor T cells via transfection and synergism with ICI (186). The existing literature presents a comprehensive analysis of the recently discovered gene TNFRSF4, encompassing its clinical effectiveness and potential correlation with immune cells (187). Although the activation of TNFRSF4-signaling in leukemic stem cells did not lead to the reduction of regulatory T cells, it impeded the ability of regulatory T cells to protect leukemic stem cells against CD8+ cytotoxic T lymphocyte-induced death. The levels of TNFRSF4 mRNA were notably elevated in the bone marrow of individuals recently diagnosed with chronic myeloid leukemia. These levels were found to be linked to the expression of the transcription factor FOXP3, which is specifically restricted to regulatory T cells (188). A series of research studies have explored the role of TNFRSF4 as a therapeutic agent in preclinical tumor models, highlighting its significant contribution to immunotherapy (189, 190). Recent findings indicate that TP53 mutations enhance the immunogenicity of breast cancer, and there is a correlation between elevated TNFRSF4 expression and TP53 mutations (191). Conversely, in patients with relapsed acute myeloid leukemia, TNFRSF4 expression is significantly increased in CD8+ T cells and regulatory T cells (Tregs) when compared to healthy donors (192).TNFRSF4 has been identified as a novel molecule in endometrial cancer (EC), and its increased expression is associated with favorable clinical outcomes. This elevated expression is closely linked to the abundance of CD4+ and CD8+ T cells. As a secondary immune checkpoint, TNFRSF4 has potential implications for endometrial cancer patients’ prognosis and immunomodulation (193). We previously introduced a novel prognostic model that significantly predicted survival outcomes based on TNFRSF4 and its association with age. TNFRSF4 exhibited a noteworthy survival association and negative correlation with age. Senescence and immunological-related pathways were highly correlated with TNFRSF4 expression (19). Given its significance in prediction and therapy, clinical agents aimed at TNFRSF4 present a promising avenue for managing tumor progression. This approach becomes increasingly attainable with the advent of newly developed direct and/or broad-spectrum small molecule inhibitors targeting checkpoint proteins. However, further research is needed, especially in the context of elderly patients.

### 2.11 TNFSF15

Tumor necrosis factor ligand superfamily member 15(TNFSF15), also known as TNF-like ligand 1A (TL1A) or vascular endothelial growth inhibitor (VEGI) which belongs to the TNF ligand family and is stimulated by TNF and IL-1α. It serves as an immune checkpoint gene, regulates the immune system, maintains cellular viability, and modulates inflammatory responses (194). The expression of the TNFSF15 protein is predominantly observed in immune cells, including dendritic cells, monocytes, and T cells, and has been associated with diverse immune-mediated mechanisms (195). The TNFSF15 gene interacts with its corresponding receptor, death receptor 3 (DR3) or TNFRSF25, which exhibits a preponderant expression on T cells (194). This interaction facilitates the transmission of signals that exert an impact on T-cell proliferation, cytokine production, and differentiation.

Preclinical models of autoimmune diseases and cancer have demonstrated promising results in blocking the TNFSF15-DR3 pathway, indicating that targeting TNFSF15 could potentially mitigate abnormal immune responses and alleviate inflammation. Maintaining vascular and lymphatic vessel homeostasis, especially in the presence of diseases such as cancer and stroke, relies heavily on its pivotal function, making it a plausible therapeutic target (196–199).

TNFSF15 is a cytokine that can inhibit the formation of new blood vessels and may also stimulate the development and specialization of macrophages into the M1 phenotype, which is known for its ability to destroy tumors. Scientists discovered (200) that the development of Lewis lung carcinoma cells in mice was significantly slowed when those cells had higher levels of TNFSF15. Further, TNFSF15 may simultaneously decrease the expression of VEGFR1 on the cell membrane and increase the synthesis of soluble VEGFR1, shifting the VEGF/VEGFR1 signaling pathway from promoting angiogenesis to inhibiting angiogenesis (200). Our team previously revealed that TNFSF15 gene expression was downregulated in clear cell renal cell carcinoma of geriatric with negative association between the TNFSF15 gene expression and age (59). Moreover, the protein-protein interaction between TNFSF15, other immune checkpoints, and proteins associated with aging demonstrates TNFSF15’s involvement in the aging mechanism. Furthermore, it has been established that the TNFSF15 gene is involved in several biological processes, such as senescence, cellular senescence, immune system, immune cell infiltration, and immunological function. An additional investigation demonstrated an age-dependent correlation of TNFSF15, which was observable not solely in individuals with cancer but also in those with other ailments related to the immune system (201, 202). It’s important to note that the role of TNFSF15 in immunotherapy is still an active area of investigation, and its significance may vary depending on the cancer type and individual patient characteristics (203–206). As our understanding of TNFSF15’s role in the immune response and its interactions with immunotherapies continues to evolve, it may become a more prominent factor in tailoring personalized treatment strategies, particularly in the elderly group.

### 2.12 VSIG4

V-set and Ig domain-containing 4(VSIG4), also known as CRIg or Z39Ig is a newly discovered B-7 related protein (207). The VSIG4 molecule serves as a receptor for complement C3, facilitating the elimination of pathogens. Additionally, it possesses the ability to impede the proliferation of CD4+ and CD8+ T-cells, as well as the production of IL-2, through the binding of an unknown receptor on T-cells (208, 209). The mRNA of VSIG4 exhibits significant expression levels across multiple tissues, with notable prevalence in the liver, lung, placenta, and potentially the central nervous system. However, the protein manifestation of VSIG4 is limited to the macrophage surface (209). According to recent research, VSIG4+ cells have been detected in the tissues of individuals with rheumatoid arthritis, atherosclerosis, and chronic hepatitis B virus infected livers, implying that VSIG4 may serve as a contributing factor to the development of such inflammatory conditions (210). The protein VSIG4 has garnered attention in inflammatory disease and cancer due to its multifaceted checkpoint properties. Additional research conducted *in vitro* and *in vivo* has shown that anti-VSIG4 antibodies can repolarize M2 macrophages and trigger an immunological response that ultimately activates T cells (211). Upregulating VSIG4 enhanced the production of pro-inflammatory cytokines in M2 macrophages as well as pro-inflammatory cytokines originating from myeloid cells and T cells (211). VSIG4 has been identified as a novel biomarker of aging macrophages in tissues that are linked to age-related systemic inflammation and immunosenescence (212). The accumulation of VSIG4 in male mice exhibited a significant correlation with heightened physiological frailty, suggesting its connection to a fundamental mechanism of the natural aging process of organisms (212). Upregulating VSIG4 enhances the production of pro-inflammatory cytokines in M2 macrophages as well as pro-inflammatory cytokines originating from myeloid cells and T cells (212). Researchers have discovered that gut microbial DNA and the immunological checkpoint gene VSIG4 play crucial opposing roles in both healthy aging and the development of hypertension and diabetes associated with aging (213). VSIG4 also has a vital function in promoting “healthy aging” by inhibiting insulin resistance and hypertension, which are often linked to the aging process. Although the exact role of VSIG4 in advancing cancerous growth is not yet fully understood, recent research has shown that VSIG4 promotes cell proliferation, migration, and resistance to immune attacks. This identifies VSIG4 as a potential target for cancer treatment, particularly for patients with a positive prognosis (214, 215). In summary, VSIG4 shows promise as a potential therapeutic target for ICI in cancer therapy (216). However, it’s crucial to emphasize that ongoing research is investigating the role of VSIG4 in the response to ICI, and its significance may differ depending on the specific cancer type and patient groups. Further studies are necessary to gain a deeper understanding of how VSIG4 expression impacts ICI responses and to explore the potential of combining VSIG4-targeted therapies with ICI. Consequently, the clinical utility of VSIG4 as either a predictive biomarker or a therapeutic target in immunotherapy is a field in continual development including older patients.

## 3 Conclusion and perspectives

Tumors derived from individuals belonging to distinct age groups exhibit varying responses to ICI, which may be attributed to the expression profile of age-related immune genes. This observation accompanies the documented rise in cancer occurrence and mortality with advancing age (32). However, only anecdotal evidence suggests that elderly individuals treated with PD-1, PD-L1, and CTLA-4 antibodies do not have inferior responses or have increased toxicity as a consequence of their advanced age, which is encouraging (217, 218). Still, the age issue will become critical as new medicines are approved and novel combinations are explored; broader and more extensive studies concentrating on the age question will become increasingly important, and they should be considered when conducting clinical trials. It’s crucial to note that not all genes associated with aging hold the potential to serve as viable targets. The attainment of satisfactory efficacy relies on combining these genes with an adaptive operational strategy and efficient carriers. Moreover, certain age-related genes may function solely as prognostic indicators or have the capability to identify effective medications for specific diseases (219–222). It is also vital to have a strong synergy with other genes and signaling pathways to prevent excessive signaling cascades (223, 224) that will lead to uncontrolled, unfavorable effects.

The secretion of SASP by cancer cells in a state of senescence attracts immune cells and inhibits the proliferation of neighboring tumor cells. Nevertheless, the persistent and prolonged buildup of SASP would facilitate the proliferation of tumors and their spread to other parts of the body (225). Hence, an optimal approach for eradicating malignant cells can include a two-step process: senescence-inducing treatment and senolytic therapy (226–228). Senescence of malignant cells can be induced by a variety of mechanisms, including chemotherapeutic agents (e.g., inhibitors of topoisomerase I and II, platinum-based therapy, alkylating agents, and microtubule inhibitors), upregulation of cyclin-dependent kinases (CDK), telomere attrition, and epigenetic region modulation (227, 229–239). Moreover, CDK inhibitors, including those for CDK4/6, CDK2/4/6, CDK7, and CDK12, are capable of halting the progression of malignant cell cycles in various types of cancer (240–243). Recent research has also demonstrated that cancer cell senescence can be increased by inhibiting telomerase (e.g., with the inhibitors GRN163L and BIBR15), DNA methyltransferase (decitabine), and histone deacetylase (GCN5, PCAF, p300/CBP, and Tip60) (232, 244–247). We expect further trials and products to emerge in the aging process, contributing to a comprehensive understanding of targetability.

**Figure 5:**
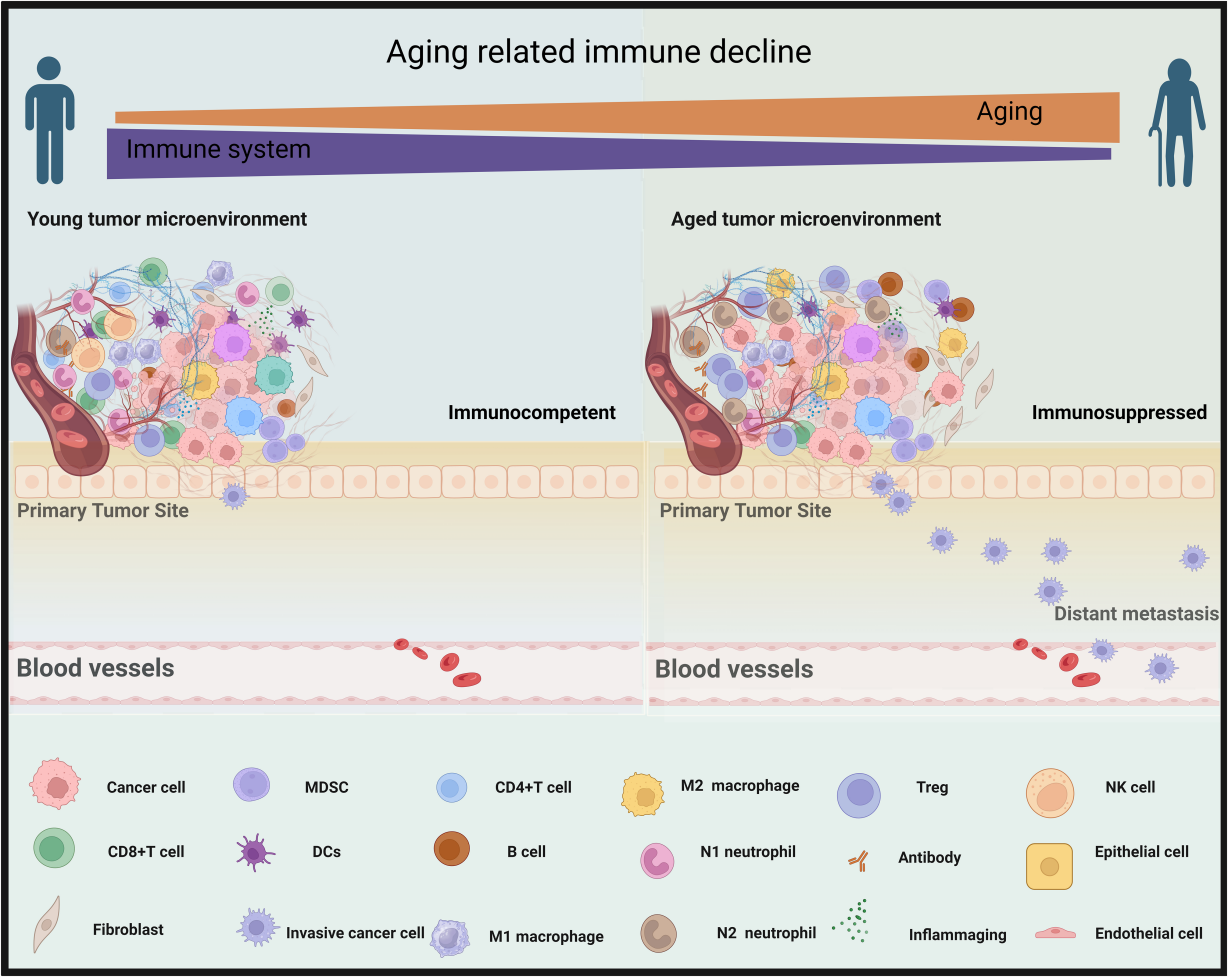
Aging contributes to a decline in immune function within tumor microenvironments. In the left panel, the tumor microenvironment of a young person is immunocompetent, with an abundance of cancer-fighting immune responses (CD8+T cells, M1 macrophages, natural killer (NK) cells, CD4+ T cells, N1 neutrophil, and dendritic cells [DCs]) that counteract the cancer growth and metastasis. Right, elderly cancer patients, as depicted in the right panel, exhibit an immunosuppressed microenvironment with a prevalence of less effective immune components (M2 macrophages, myeloid-derived suppressor cells (MDSC), B cells, T regulatory T cells [Treg], N2 neutrophils, and (inflammaging which is an age-related increase in the levels of pro-inflammatory markers in blood and tissues), that are less equipped to combat metastasizing cancer cells effectively. By BioRender (https://app.biorender.com/).

Immunosenescence, a process characterized by the immune system’s adaptive responses to previous challenges encountered over an individual’s lifetime, has been associated with unfavorable clinical outcomes (18, 248, 249). In circumstances unrelated to tumors, it is commonly observed that older people tend to exhibit less efficient immune responses when facing diseases like the new pandemic due to coronavirus disease 2019 (COVID-19) (4, 250). This phenomenon is typically linked to the systemic immune senescence. Specifically, age-related immune alterations, such as a reduction in TCR diversity, a decline in the cytotoxic cell’s capability, and an increase in inflammatory signaling, and others (**Figure 5**) (251–254).

The aforementioned findings highlighted the potential importance of immunological aging and its effect on malignancy genesis and progression (255). However, in order to apply this knowledge to cancer treatment and patient management, it is necessary to conduct a more thorough analysis of the relationship between the overall immune system and the specific immunological milieu within tumors, which is influenced by the aging process, especially in the ICI era. Despite the often observed reduction in immune function that accompanies the aging process, a significant body of evidence from clinical trial analysis indicates that older individuals derive comparable or even enhanced benefits from ICI therapy when compared to their younger counterparts (62, 68, 256, 257). In reconciling inconsistencies and establishing a consensus regarding the impact of age on immunotherapy response, recent studies indicated that age-related impairments in the immune system did not influence the efficacy and toxicity of ICI in geriatric patients compared to their younger counterparts (217, 258–263). Therefore, regardless of chronological age, ICI can be considered for elderly patients. Even so, there remains ongoing debate regarding this matter, and it is worth noting that older individuals with cancer may receive less frequent utilization of ICI compared to young cancer patients (62, 264, 265).

In summary, aging and cancer are closely intertwined, as aging is a risk factor for the development of cancer. Nevertheless, advancements in cancer treatment, particularly ICI therapy, offer new hope for patients by leveraging the body’s immune system’s ability to overcome cancer. Continued research and innovation in this field are crucial to improving patient outcomes, expanding the application of ICI, and ultimately finding more effective and personalized approaches to combating cancer in the context of aging.

## Author contributions

AA: Validation, Visualization, Writing – original draft, Writing – review & editing. MS: Conceptualization, Visualization, Writing – original draft. YJ: Validation, Visualization, Writing – review & editing. LY: Investigation, Writing – original draft, Writing – review & editing. SW: Project administration, Supervision, Writing – review & editing. XZ: Data curation, Investigation, Writing – original draft. QC: Funding acquisition, Validation, Writing – review & editing. KY: Visualization, Writing – review & editing. JZ: Conceptualization, Resources, Writing – review & editing. DY: Project administration, Resources, Supervision, Writing – review & editing.

## Glossary

**Table:**

TIICs	tumor-infiltrating immune cells
ICI	immune checkpoint inhibitors
PD-L1	programmed death-ligand 1
PD-1	programmed death 1
CTLA-4	cytotoxic T lymphocyte antigen 4
PD-L2	programmed death-ligand
CD80	cluster of differentiation 80
JAK2	janus kinase 2
LAG3	lymphocyte activation gene 3
HAVCR2	hepatitis A virus cellular receptor 2
TGF-β	transforming growth factor-β
CXCL9	C-X-C motif chemokine ligand 9
CD86	cluster of differentiation 86
TILs	tumor-infiltrating lymphocytes
Tim-3	mucin-domain containing-3
IDO	indoleamine 2,3-dioxygenase
PPI	protein-protein interactions
APOBEC	apolipoprotein B mRNA editing enzyme, catalytic polypeptide-like family
IDH1	isocitrate dehydrogenase 1
ATRX	alpha thalassemia/mental retardation syndrome X-linked gene
TP53	tumor protein p53
GATA3	GATA binding protein 3
CDH1	cadherin 1
ARID1A	AT-rich interaction domain 1A
KRAS	KRAS proto-oncogene,GTPase
BRAF	B -Raf proto -oncogene, serine/threonine kinase
HER2+	human epidermal growth factor receptor 2-positive
TERT	telomerase reverse transcriptase
KEGG	kyoto encyclopedia of genes and genomes
GO	gene ontology
CD274	cluster of differentiation 274
NF-κB	nuclear factor-κB
MAPK	mitogen-activated protein kinase
mTOR	molecular target of rapamycin
STAT	signal transducer and activator of transcription
c-myc	cellular myelocytomatosis
n-Sen	nutlin3a-induced senescent cells
d-Sen	damage-induced senescent cells
SASP	senescence-associated secretory phenotypes
TIGIT	T cell immunoreceptor with immunoglobulin and ITIM domain
MHC	major histocompatibility complex
HLA-DR	MHC II cell surface receptor
AD	alzheimer’s disease
CNS	central nervous system
Th1	T helper 1
Ig	immunoglobulin
Tc1	T cytotoxic 1
IFN-γ	interferon gamma
mAbs	monoclonal antibodies
HLH	hyperactivated T and myeloid cells, hemophagocytic lymphohistiocytosis
SPTCL	subcutaneous panniculitis-like T-cell lymphoma
ICOS	inducible costimulator
KLRG-1	killer cell lectin-like receptor G1
TCR	T cell receptor
Gal-9	galectin-9
GBMLGG	glioblastoma and low-grade glioma
LUAD	lung adenocarcinoma
PRAD	prostatic adenocarcinoma
SARC	sarcoma
NSCLC	non–small cell lung cancer
HNSCC	head and neck squamous cell carcinoma
ACC	adenoid cystic carcinoma
TGFB1	transforming growth factor beta 1
TME	tumor microenvironment
RECIST	response evaluation criteria in solid tumors
Tif1γ	transcriptional intermediary factor 1
ALK	activin receptor-like kinase
TMB	tumor mutational burden
PI3K	phosphoinositide 3-kinase
iAge	inflammatory aging clock
MPNs	myeloproliferative neoplasms
RET	rearranged during transfection
B2M	b-2-microglobulin
TNFRSF4	tumor necrosis factor receptor superfamily member 4
TRAF2	tumor necrosis factor receptor-associated factor 2
AML	acute myeloid leukemia
EC	endometrial cancer
Tregs	regulatory T cells
TNFSF15	tumor necrosis factor ligand superfamily member 15
TL1A	TNF-like ligand 1A
VEGI	vascular endothelial growth inhibitor
DR3	death receptor 3
ccRCC	clear cell renal cell carcinoma
LLC	lewis lung carcinoma
VSIG4	V-set and Ig domain-containing 4
AS	atherosclerosis
HBV	chronic hepatitis B virus
COVID-19	coronavirus disease 2019
NK	natural killer
MDSC	myeloid-derived suppressor cells
CDK	cyclin-dependent kinases
