# Supplementary material for: Aging-related biomarker discovery in the era of immune checkpoint inhibitors for cancer patients
Source: Front Immunol. 2024 Mar 15;15:1348189. doi: 10.3389/fimmu.2024.1348189 (PMC11000233; doi:10.3389/fimmu.2024.1348189)
Supplement: Supplementary file 1 [file DataSheet_1.docx]

**Supplementary table1 . Predominant (Gene Ontology) GO processes for immune biomarkers with aging.**

| Category | Term | p_value | fdr | Description |
| --- | --- | --- | --- | --- |
| Process | GO:0050868 | 2.76E-15 | 8.85E-12 | Negative regulation of T cell activation |
| Process | GO:0042130 | 1.26E-14 | 2.06E-11 | Negative regulation of T cell proliferation |
| Process | GO:0051249 | 1.12E-14 | 2.06E-11 | Regulation of lymphocyte activation |
| Process | GO:0006955 | 1.03E-12 | 6.60E-10 | Immune response |
| Process | GO:0002683 | 1.30E-12 | 7.61E-10 | Negative regulation of immune system process |
| Component | GO:0009897 | 8.88E-12 | 8.07E-09 | External side of plasma membrane |
| Component | GO:0009986 | 4.75E-12 | 8.07E-09 | Cell surface |
| Component | GO:0005886 | 1.58E-06 | 0.00067 | Plasma membrane |
| Component | GO:0016020 | 4.60E-05 | 0.013 | Membrane |

**
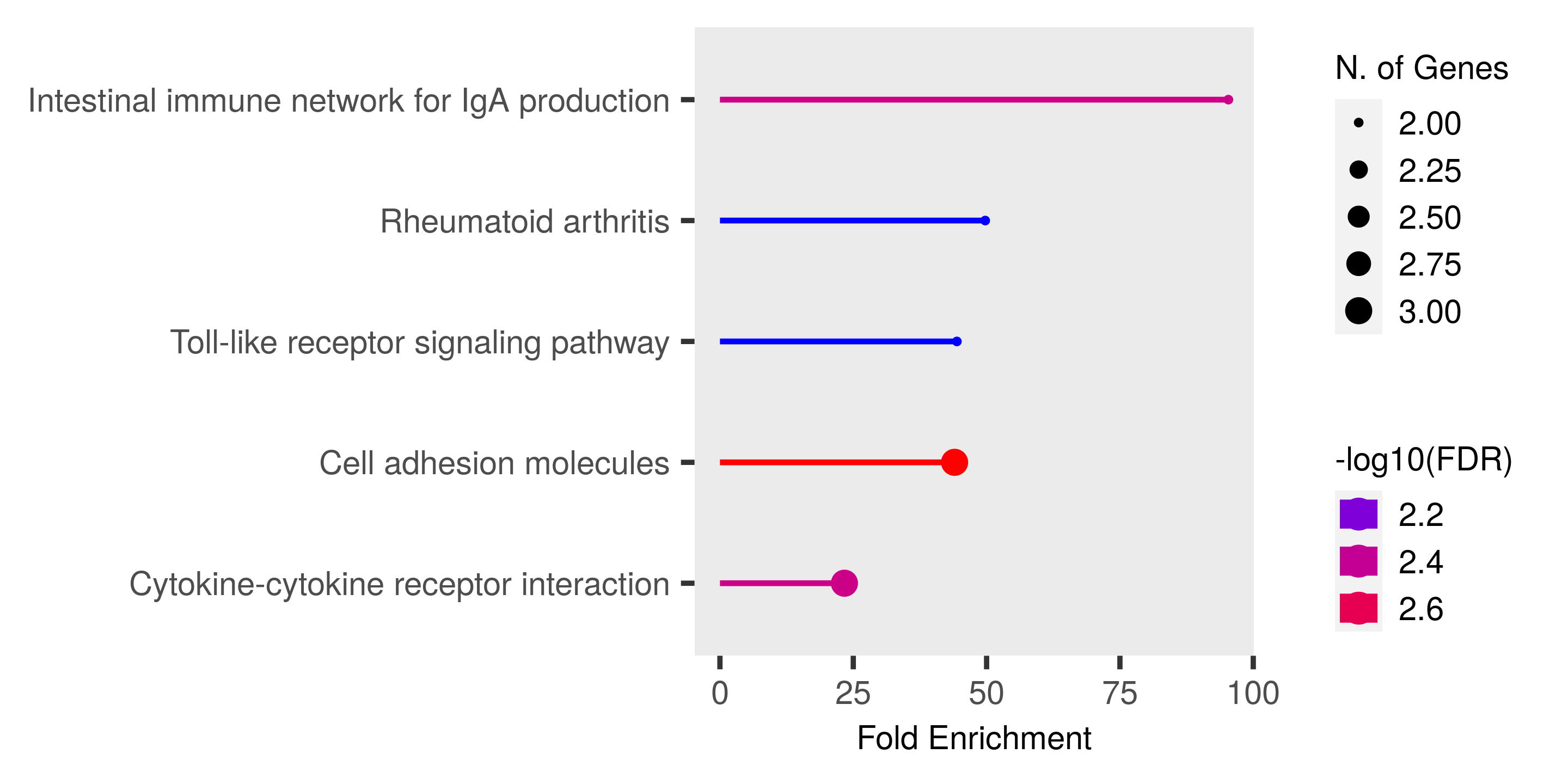
**

**Supplementary Figure1 .**

The Kyoto Encyclopedia of Genes and Genomes pathways (KEGG) enrichment pathways for selected immune biomarkers with aging.
